# Simple, efficient and accurate method toward the monitoring of ethyl butanoate traces

**DOI:** 10.1007/s11082-021-03497-4

**Published:** 2022-01-22

**Authors:** Z. A. Alrowaili, Hussein A. Elsayed, Ashour M. Ahmed, T. A. Taha, Ahmed Mehaney

**Affiliations:** 1 Physics Department, College of Science, Jouf University, P.O. Box: 2014, Sakaka, Saudi Arabia; 2 TH-PPM Group, Physics Department, Faculty of Science, Beni-Suef University, Beni-Suef, 62512 Egypt; 3 Physics Department, Faculty of Science, Beni-Suef University, Beni-Suef, 62512 Egypt

**Keywords:** Dry exhaled breath, Ethyl butanoate, TP resonance, Photonic crystals, Biomarkers and biosensors, Defect mode

## Abstract

We introduce in this research a simple, accurate, safe, and efficient design for the detection of ethyl butanoate that be present in the dry exhaled breath. In particular, the presence of ethyl butanoate in the dry exhaled breath could be utilized as a platform for the diagnosing of COVID 19. The main idea of this theoretical investigation is based on the inclusion of a cavity layer between a thin layer of Au and the well-known one-dimension photonic crystals. Accordingly, the cavity layer is filled with dry exhaled breath. The numerical results are investigated in the vicinity of the Drude model and transfer matrix method. The investigated results show the appearance of Tamm plasmon resonance in the reflectance spectrum of our design through the IR region. Such resonant mode provides very high sensitivity with the change in the concentration of ethyl butanoate. We have examined the performance of the proposed sensor by calculating its sensitivity, detection limit, detection accuracy, quality factor and figure of merit. The designed sensor could receive sensitivity of 0.3 nm/ppm or 260,486 nm/RIU, resolution of 7 ppm and quality factor of 969.

## Introduction

In recent times, many researchers devoted their attention to new types of biosensors such as plasmonic photothermal (PPT), electrochemical (EC) and field-effect transistor (FET) (Qiu et al. 2020; Seo et al. 2020; Mahari et al. 2020). Walking over these steps, we have aimed to present a novel design for the detection of ethyl butanoate traces in exhaled breath based on the well-known one-dimensional photonic crystals (1DPCs). Specifically, the exhaled breath of COVID-19 patients could contain higher levels of ethyl butanoate (Chen et al. 2020).

PCs present a new class of periodic structures in which the periodicity of refractive index among one, two, or three dimensions plays the main role in many promising applications (Mehaney et al. 2021; Abadla et al. 2021, 2020a; Zhang and Lv 2020). Whilst PCs could be designed or fabricated due to the sticking of two materials at least with different optical characteristics (Peng et al. 2017; Chen et al. 2019a). This hypothesis could be leading in the control of electromagnetic (EM) waves' propagation and photon localization as well. The control of EM propagation comes as a result of the appearance of some stop frequency bands that coincide in their values with those of the incident EM waves (Abadla et al. 2020b; Razi and Ghasemi 2019; Sancho-Fornes et al. 2019). Such frequency bands were later granted the name photonic band gaps (PBGs). On the other hand, photon localization can be investigated as the periodicity of the PC structure has been broken (Aly and Elsayed 2012; Ramanujam et al. 2020).

In this context, PCs characteristics opened the road towards a massive number of applications such as gas and temperature sensing (Chen et al. 2019a; Abadla et al. 2020b), optical integrated circuits (Razi and Ghasemi 2019), limiters (Vasudevan et al. 2019), couplers (Hameed et al. 2009), polarization rotators (Hameed et al. 2013) and optical filters (Trabelsi et al. 2019; Sadykov et al. 2019). Recently, the inclusion of PCs in biosensing applications received promising attention in the detection and monitoring of immunosensing (Sancho-Fornes et al. 2019), bacteria (Ramanujam et al. 2020), protein (Chen et al. 2019b), hemoglobin (Abadla and Elsayed 2020), creatinine (Aly et al. 2020), blood plasma (Bijalwan et al. 2021) and cancer diseases (Bijalwan et al. 2021; Chakravarty et al. 2013; Sinibaldi et al. 2018; Mollah et al. 2020). Moreover, PC biosensors based on volatile organic compounds (VOCs) from exhaled breath could be of potential interest especially, since the exhaled breath of healthy persons contains more than 874 types of VOCs (Schwoebel et al. 2011; Jalal et al. 2018). Isoprene, ethanol, methanol, acetone and fewer compounds like aldehydes pentane, alcohols and ketones represent the most common VOCs (Jalal et al. 2018; Fenske and Paulson 1999; Amann et al. 2014). Thus, the abnormal concentration of these VOCs through the exhaled breath expresses significant indicators for many diseases such as lung diseases (Boots et al. 2014), liver diseases (Tangerman et al. 1983), lung cancer (Szulejko et al. 2010) and schizophrenia (Popa et al. 2015).

In this study, we intend to present a simple and safe biomarker for the early detection of COVID 19 based on 1D PCs. Here, we have designed a cavity layer to be sandwiched between a metallic layer of Au and the proposed 1D PCs. The essential idea of our work is based on the appearance of Tamm plasmon (TP) resonance inside the PBG. Then, the detection procedure can be investigated based on the shift in TP resonance peak due to the change in the concentration of ethyl butanoate from 0 to 100 ppm through the dry exhaled breath. In particular, the cavity layer is designed to fill with dry exhaled breath. In fact, the appearance of TP resonance through 1D PCs is widely investigated both theoretically and experimentally for many sensing applications (Mehaney et al. 2021; Ahmed and Mehaney 2019; Chiang et al. 2004; Auguié et al. 2014; Zaky et al. 2020). In particular, the experimental verification of cavities through the periodic multilayer structures and PCs as well received considerable attention. In this context, Guan et al. (2011) fabricated a dual-wavelength vertical cavity surface emitting laser with asymmetric 1D PCs by using a metal–organic chemical vapor deposition (MOCVD) system. Moreover, Cabrini et al. (2006) prepared Si_3_N_4_/SiO_2_ periodic structure as 1D photonic with micro-wide cavity using ion beam (FIB) lithography and low-pressure chemical vapor deposition (LPCVD) technique. Also, Stinson et al. (2021) investigated PCs with a defect fabricated based on the two-photon polymerization. In addition, high sensitivity values are expected for this purpose. The transfer matrix method (TMM) and the optical properties of the constituent materials express the theoretical formalism of our study. The numerical results discuss the shift in TP resonance with the change of ethyl butanoate concentration. Moreover, we have investigated the parameters affecting the performance of the proposed sensor such as, sensitivity, detection limit, the figure of merit, quality factor and uncertainty.

## Sensor design and theoretical formalism

**Fig. 1:**
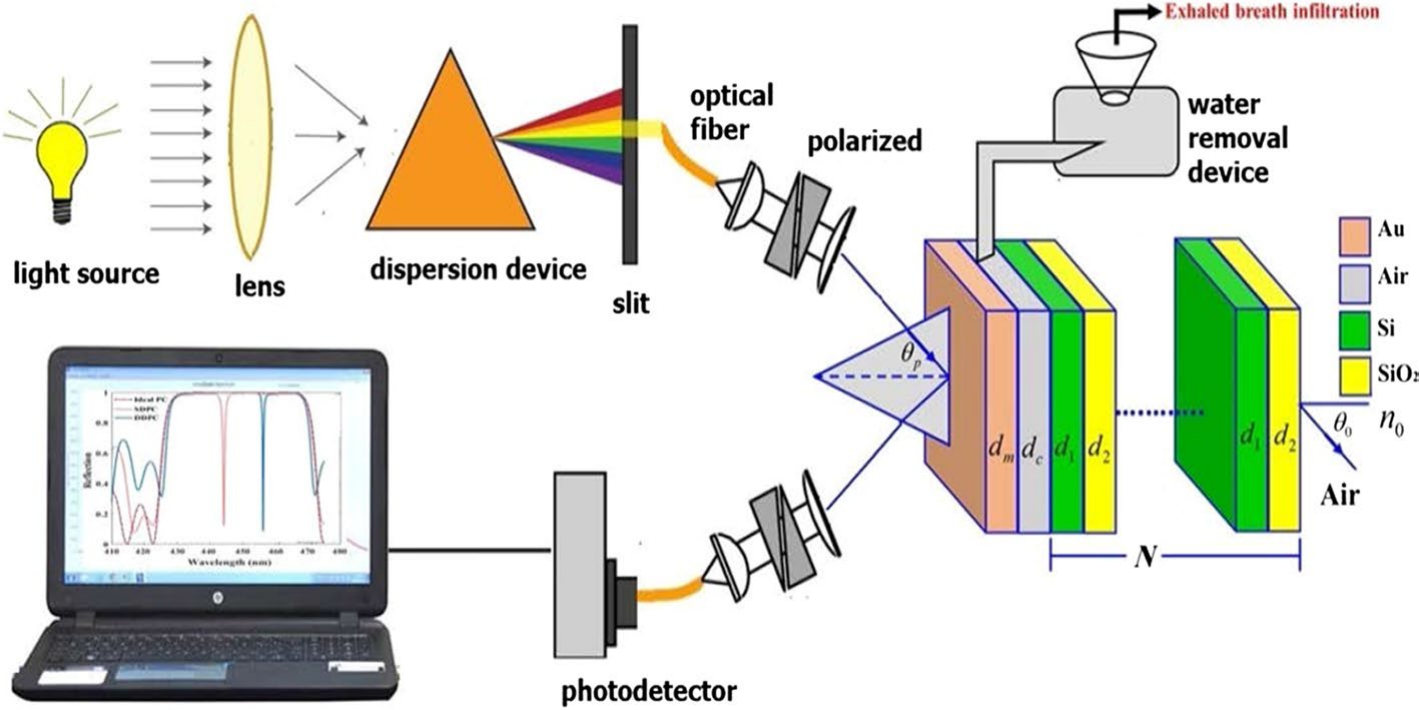
The schematic description of the proposed ethyl butanoate 1D PCs sensor in which a cavity layer from the air of thickness d_c_ is sandwiched between a thin layer of Au with thickness d_m_ and the 1D PCs. Here, the 1D PCs are designed from N unit cells of Si and SiO_2_ of thicknesses d_1_ and d_2_, respectively

In this section, we have discussed the sensor design and the basic equations for the theoretical formulation as well. Figure 1 shows the optical setup of the proposed sensor technique that contains two essential parts. The first one contains the essential design of the 1D PCs configurations in which a cavity layer is sandwiched between a thin metallic layer of Au and the 1D PCs. 1D PCs are designed from 10-unit cells. Each unit cell contains two layers Si and SiO_2_ with thicknesses (d_1_ and d_2_) and refractive indices (n_1_ and n_2_), respectively. Here, the cavity is designed to receive the dry exhaled breath to detect the concentration of the ethyl butanoate. Then, the second part of our design is consisting of the optical system. The optical system divides into input and output signals. For the input signal, a light beam is emitted from a light lamp containing wavelengths in the range from 2 to 6 μm to focusing by a lens through the optical fiber transmission. Then, the collimated light is passing through a dispersive device (like a prism) and polarizer to select the monochromatic wavelength and the mode of polarization (TE polarization in our study) as well. Here, the light source is placed on a rotate table to change the incident angle of light. Next, the light beam is directed towards the lateral face of the prism of the proposed structure [prism/Au/ cavity/1D-PC]. In this regard, the exhaled breath sample is dried to remove water vapor and collected in the cavity for analysis. The reflected light on the other side of the prism is collected and analyzed by the photodetector spectrometer. Finally, the spectrum appears on a laptop screen. A sharp dip of intensity on the reflected light beam can be observed for Tamm plasmon resonance. All steps are carried out at room temperature.

Therefore, the TMM method is a simple and efficient method to describe the response of the incident waves through periodic structures like 1D PCs (Abadla et al. 2021; Peng et al. 2017; Chen et al. 2019a; Razi and Ghasemi 2019; Aly and Elsayed 2012; Ramanujam et al. 2020; Born and Wolf 1999). Hereinafter, we have considered y as the direction of propagation for the incident EM waves. Thus, through a specified layer *j*, the matrix representation of the electric and magnetic field’s components are given as (Orfanidis 2008; El-Naggar 2015):

1 $$\left( {\begin{array}{*{20}c} {E_{j} } \\ {H_{j} } \\ \end{array} } \right)\, = \,\left( {\begin{array}{*{20}c} {\exp \,( - ik_{j} y)} & {\exp \,(k_{j} y)} \\ {n_{j} \exp \,( - ik_{j} y)} & { - n_{j} \exp \,(ik_{j} y)} \\ \end{array} } \right)\,\left( {\begin{array}{*{20}c} {C_{j} } \\ {F_{j} } \\ \end{array} } \right),$$

where (*C*_*j*_ and *F*_*j*_) represent the amplitudes of the fields through the specified layer and (*k*_*j*_ and *n*_*j*_) describe the wave vector and the refractive index, respectively. Then, the fields components at the interface between two consecutive layers is given as,

2 $$\left( {\begin{array}{*{20}c} {E_{j} } \\ {H_{j} } \\ \end{array} } \right)\, = \,\left( {\begin{array}{*{20}c} {\cos \,(k_{j} \upsilon_{j} )} & {\left( {\frac{ - i}{{\delta_{j} }}} \right)\sin \,(k_{j} \upsilon_{j} )} \\ { - i\delta_{j} \sin \,(k_{j} \upsilon_{j} )} & {\cos \,(k_{j} \upsilon_{j} )} \\ \end{array} } \right)\,\left( {\begin{array}{*{20}c} {E_{j + 1} } \\ {H_{j + 1} } \\ \end{array} } \right)\, = \,t_{j} \left( {\begin{array}{*{20}c} {E_{j + 1} } \\ {H_{j + 1} } \\ \end{array} } \right),$$

with $\upsilon_{j} \, = \,d_{j} \cos \theta_{j} \,$, $\delta_{j} \, = \,\frac{{\left( { - 1} \right)^{\alpha } \mu_{j}^{\alpha - 1} k_{j} \,\cos \theta_{j} }}{{\varepsilon_{j}^{\alpha } \omega }}$ with *α* = 0, 1 for TE and TM polarizations; respectively and $\theta_{j}$ is the angle of incidence through layer *j*. Therefore, the response of the electric and magnetic fields through our designed structure is described as,

3 $$T\, = \,\left( {\begin{array}{*{20}c} {T_{11} } & {T_{12} } \\ {T_{21} } & {T_{22} } \\ \end{array} } \right)\, = \,\left( {t_{Au} } \right)\left( {t_{cavity} } \right)\left( {t_{Si} t_{{SiO_{2} }} } \right)^{10}$$

Making use of the matrix elements of Eq. (3), we can describe the reflectivity of our design as:

4 $$R\, = \,\left| {\frac{{\left( {T_{11} + T_{12} \delta_{0} } \right)\delta_{p} - \left( {T_{21} + T_{22} \delta_{0} } \right)}}{{\left( {T_{11} + T_{12} \delta_{0} } \right)\delta_{p} + \left( {T_{21} + T_{22} \delta_{0} } \right)}}} \right|^{2} ,$$

where *δ*_*0*_ and *δ*_*p*_ are holding for a prism as the starting medium and air as substrate, respectively. By the end of this section, we can describe the refractive index of Au metallic thin layer based on the Drude model as Alabastri et al. (2013),

5 $$n_{Au} \, = \,\sqrt {\varepsilon_{Au} } \, = \,\sqrt {1 - \,\left( {\frac{{\omega_{p}^{2} }}{{\omega^{2} + i\gamma \omega }}} \right)} ,$$

here *ω*_*p*_ and *γ* represent the plasmon frequency of the metal and the damping constant, respectively. In the case of Au, the plasmon frequency is equivalent to 8.55 eV and the damping frequency = 0.0184 eV (Estrin et al. 2015).

## Numerical results and discussions

Herein, we present sufficient details about the investigated results of our study. The numerical results are centered on the reflectivity of our design due to the interaction with the IR radiation. In this regard, we have prepared Matlab codes to simulate the interaction of the incident electromagnetic waves with our proposed design. Moreover, we have demonstrated the effect of the variation of ethyl butanoate concentration on the position of the produced TP resonance. In particular, the shift in the spectral position of TP resonance plays the main role in judging sensor performance. Here, the thicknesses of SiO_2_ and Si are set to be 900 nm and 200 nm, respectively. Then, the refractive index of SiO_2_ is wavelength dependent according to the following relation (Malitson 1965):

6 $$n_{{SiO_{2} }} = \,\sqrt {1 + \,\frac{{{0}{\text{.6961663}}\,\lambda^{{2}} }}{{\lambda^{{2}} - 0.004679}} + \frac{{{0}{\text{.4079426}}\,\lambda^{{2}} }}{{\lambda^{{2}} - 0.013512}} + \frac{{{0}{\text{.8974794}}\,\lambda^{{2}} }}{{\lambda^{{2}} - 97.934}}} ,$$

where λ defines the wavelength of the incident radiation in microns. Whilst the refractive index of Si is described by Sellmeier dispersion formula (Tatian 1984) as,

7 $$n_{Si} = \,\sqrt {1 + \,\frac{{{106684293}\,\lambda^{{2}} }}{{\lambda^{{2}} - 0.090912}} + \frac{{{0}{\text{.00304347484}}\,\lambda^{{2}} }}{{\lambda^{{2}} - 1.28766}} + \frac{{{1}{\text{.54133408}}\,\lambda^{{2}} }}{{\lambda^{{2}} - 1218816}}}$$

**Fig. 2:**
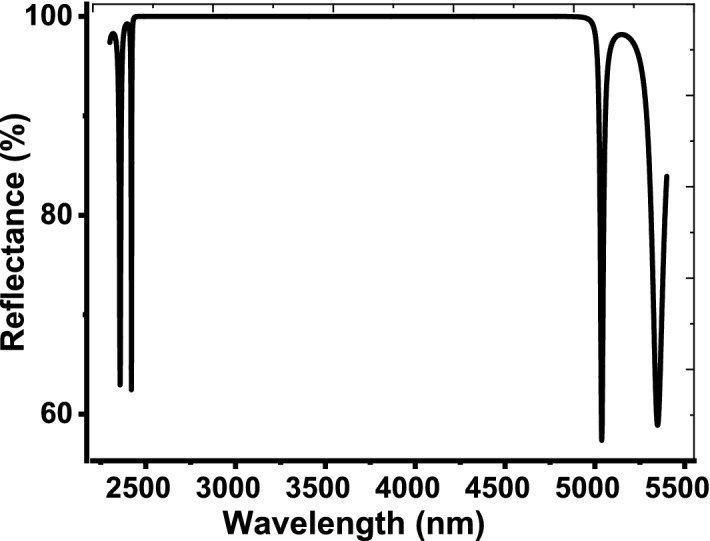
The reflectivity of the 1D PCs that configured as, [prism/(Si/SiO_2_)^10^/air] for TE mode of polarization at θ_p_ = 50°

Now, we present the investigated results of our designed sensor. Figure 2 describes the reflectivity of the 1D PCs that are configured as, [prism/(Si/SiO_2_)^10^/air] for the case of TE polarization at an angle of incidence = 50°. Actually, the choice of this specified value of the angle of incidence comes as a result of the optimization process. In particular, this value achieves the best performance of our designed sensor. In this context, the refractive index of the designed prism is set to equal 1.3. Figure 2 shows that the structure reflectivity receives unity through a wide band of wavelengths that extends from 2442 to 4921 nm. Thus, our design provides a wide PBG with a width = 2479 nm. The presence of this wide PBG is due to the high contrast in refractive index between Si and SiO_2_ layers. In addition, this wide band could be of interest in producing the TP resonance without interference with the reflectance dips.

**Fig. 3:**
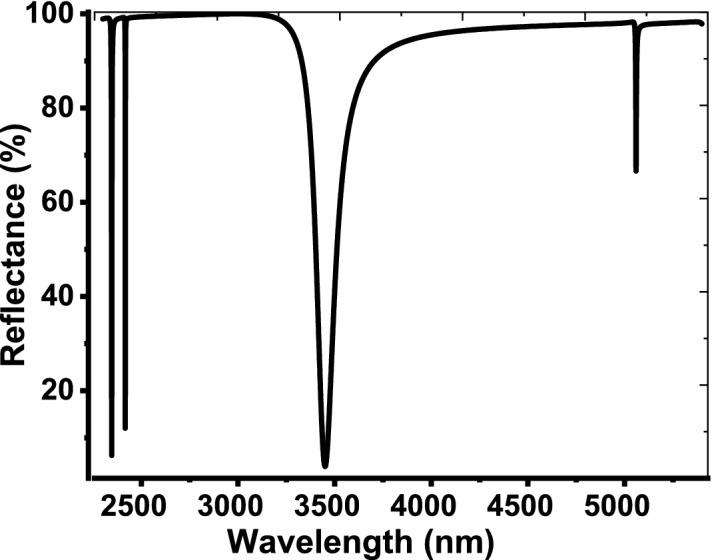
The reflectance spectrum of our design as a thin layer of Au with thickness = 10 nm is deposited over the 1D PCs

**Fig. 4:**
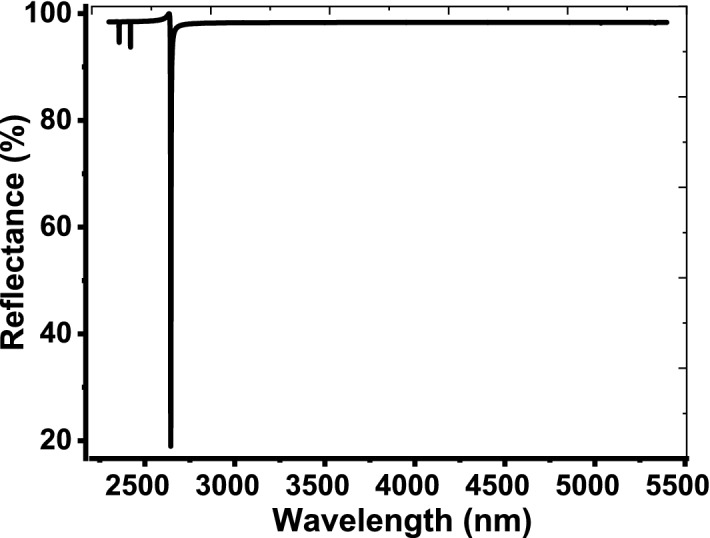
The reflectance spectrum of the 1D PCs in the presence of a thin layer of Au and cavity layer of air on its top surface

As a thin layer of Au with thickness = 10 nm is deposited over the 1D PCs, the structure reflectivity takes a different style as shown in Fig. 3. The figure clarifies the appearance of a resonant mode like the defect mode inside the PBG. This resonant mode is known as TP resonance that formed due to the oscillations of electrons at the interface between the 1D PCs and Au layer (Chiang et al. 2004; Auguié et al. 2014; Zaky et al. 2020; Guan et al. 2011; Cabrini et al. 2006; Stinson et al. 2021). TP resonance is located at 3450.4 nm with reflectivity = 3.915% and large full width at half maximum (FWHM) = 101 nm. Such resonant peak plays the main role in our detection process due to their high sensitivity for any change through the parameters of the constituent materials. By sandwiching a cavity layer from the air with thickness = 14 μm between the 1D PCs and the thin metallic layer, the designed structure is now configured as, [prism/Au/air cavity/(Si/SiO_2_)^10^/air]. Therefore, the TP resonance peak provides significant changes in its characteristics as shown in Fig. 4. Furthermore, the intensity of the reflection dips that located outside the PBG increased. In this regard, TP resonance was shifted downwards to 2644.1 nm and its reflectivity increased to 18.933%. Also, the FWHM decreased to be about 2 nm. These significant changes are due to the change in the interfacing layers at which the electrons can oscillate from Au and Si to Au and air. On other words, the change in the thicknesses or refractive indices of the interfacing layers could lead to such variations. Moreover, these changes could have significant effects on the performance of our sensor as investigated later.

**Fig. 5:**
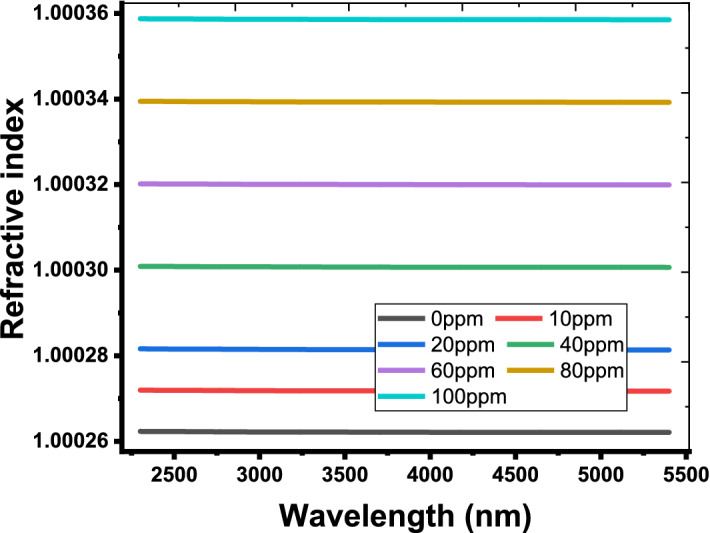
Refractive index of the dry exhaled breath at different ethyl butanoate concentrations from 0 to 100 ppm

**Fig. 6:**
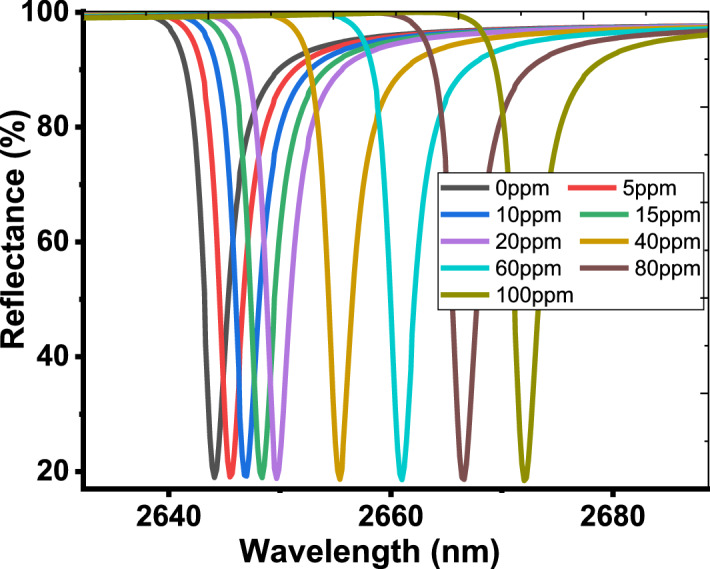
Reflectance spectrum of our designed sensor at different ethyl butanoate concentrations from 0 to 100 ppm

**Fig. 7:**
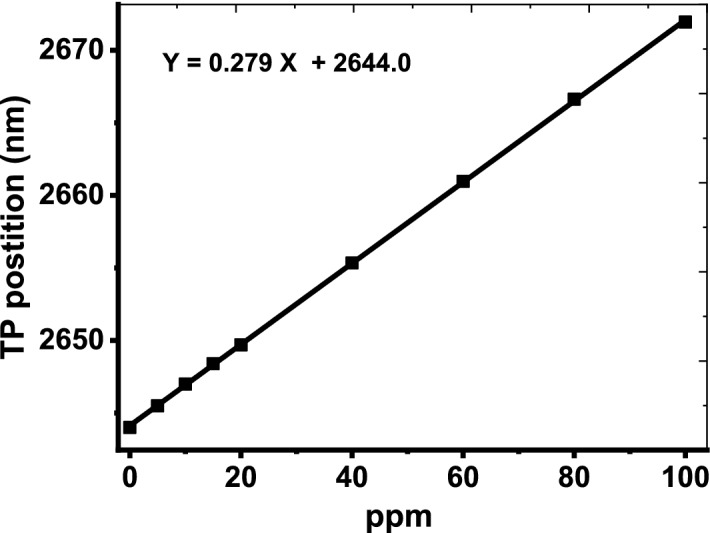
The response of TP resonance wavelength with the change in the concentration of ethyl butanoate

As the cavity layer is filled with the dry exhaled breath, the detection process can be investigated. As mentioned before, the dry exhaled breath is a combination of a large number of gases with different volume fractions. For healthy or normal persons, the dry exhaled breath contains 78% of N_2_, 16% of O_2_, 5% of CO_2_ and Ar with 1%. For others who are suffering from some diseases like lung cancer, liver diseases, lung diseases and COVID 19, some gases like toluene, isoprene and ethyl butanoate (EB) are present within the dry exhaled breath (Chen et al. 2020; Boots et al. 2014; Tangerman et al. 1983; Szulejko et al. 2010). In this context, the effective refractive index of the dry exhaled breath is depending on the refractive indices of these gases and their volume fractions as well according to the following relation (Li and Song 2010; Zhang et al. 2008; Haynes 2017; White and Fan 2008; Beheiry et al. 2010; Wu et al. 2013):

8 $$\begin{aligned} n_{eff} & = \sqrt {\sum\limits_{i} {n_{i}^{2} \,V_{i} } } \, \\ & = \sqrt {n_{{N_{2} }}^{2} \,V_{{N_{2} }} + n_{{O_{2} }}^{2} \,V_{{O_{2} }} + n_{{CO_{2} }}^{2} \,V_{{CO_{2} }} + n_{Ar}^{2} \,V_{Ar} + n_{EB}^{2} \,V_{EB} } , \\ \end{aligned}$$

where *n* and *V* represent the refractive indices and volume fractions of the different gases, respectively. Figure 5 shows the dependence of the effective refractive index on the wavelength of the incident EM waves at different values of ethyl butanoate concentrations. Figure 5 investigates that, the effective refractive index of the dry exhaled breath is almost unchanged with the variation of the wavelengths. However, its value can be changed with the increase of ethyl butanoate concentration. As the concentration of ethyl butanoate increases from 0 to 100 ppm, the effective refractive index increases from 1.000262 to 1.000358, respectively. In fact, the ability of the sensing tools based on photonic devices to detect this variation is very difficult. However, the high sensitivity of TP resonance may offer a suitable solution for this obstacle. Meanwhile, Fig. 6 describes the effect of ethyl butanoate concentration changes on the position of the TP resonance mode. As the concentration of ethyl butanoate increases to 5 ppm, the TP resonance peak is shifted upward from 2644.1 nm to 2645.5 nm. For further increase in the values of concentration to 10 ppm, 20 ppm, 40 ppm, 80 ppm and 100 ppm, the resonant peak is shifted to 2647 nm, 2649.7 nm, 2655.4 nm, 2666.6 nm and 2672 nm, respectively. Here, the TP resonance peak is shifted 27.9 nm due to the change in the concentration of ethyl butanoate 100 ppm. Such a result suggests a promising performance of our designed sensor. Moreover, the designed sensor is very sensitive to any little change in the concentration of ethyl butanoate. To sum up, we have plotted in Fig. 7, the dependence of TP position on the concentration. The figure shows that the wavelength of TP resonance varies linearly with the concentration of ethyl butanoate. This response is demonstrated according to the following relation:

9 $$\lambda_{TP} \, = \,2644\, + \,0.279\,C,$$

According to this relation, the concentration of ethyl butanoate in the dry exhaled breath can be obtained with extreme accuracy and clarity based on the wavelength values of the TP resonance peak.

Finally, we have investigated the performance of our designed sensor in the form of calculating the numerical values of some parameters such as sensitivity (S), quality factor (OF), detection limit (DL), detection accuracy (DA), sensor resolution (SR), dynamic range (DR), signal to noise ratio (SNR), the figure of merit (FM) and uncertainty (χ) as depicted in Table 1. These parameters could be defined based on the concentration of ethyl butanoate, values of TP position and its FWHM according to the following equations (White and Fan 2008; Beheiry et al. 2010; Wu et al. 2013; Naftaly and Dudley 2009; Ahmed and Shaban 2020):

10a $$S\, = \,\frac{{\Delta \lambda_{TP} }}{\Delta C}\,,$$

10b $$QF\, = \,\frac{{\lambda_{TP} }}{FWHM}\,,$$

10c $$SNR\, = \,\frac{{\Delta \lambda_{TP} }}{FWHM},$$

10d $$DR\, = \,\frac{{\lambda_{TP} }}{{\sqrt {FWHM} }}\,,$$

10e $$DA\, = \,\frac{1}{FWHM}\,,\,$$

10f $$FM\, = \,\frac{S}{\,FWHM}\,,$$

10g $$DL\, = \,\left( {\frac{\Delta C}{{1.5}}} \right)\left( {\frac{\,FWHM}{{\Delta \lambda_{TP} }}} \right)^{1.25} ,$$

10h $$SR\, = \,\left( S \right)\,\left( {DL} \right)\,,$$

10i $$\chi \, = \,\frac{{2\left( {\Delta \lambda_{TP} } \right)^{0.75} \left( {FWHM} \right)^{0.25} }}{9}$$

**Table 1:** The performance-related parameters of our designed sensor due to the concentration variation of ethyl butanoate

C (ppm)	0	5	10	15	20	40	60	80	100
λTP (nm)	2644	2645.5	2646.99	2648.4	2649.69	2655.34	2660.96	2666.62	2671.95
Intensity (%)	18.93	19.04	19.18	18.88	18.77	18.63	18.55	18.59	18.39
FWHM (nm)	2.79	2.73	2.86	2.78	2.76	2.76	2.78	2.76	2.76
S (nm/ppm)	–	0.3	0.299	0.293333	0.2845	0.2835	0.282667	0.28275	0.2795
FM (/ppm)	–	0.10989	0.104545	0.105516	0.10308	0.102717	0.101679	0.102446	0.101268
QF	947.6703	969.0476	925.521	952.6619	960.0326	962.0797	957.1799	966.1667	968.0978
DA (/nm)	0.358423	0.3663	0.34965	0.359712	0.362319	0.362319	0.359712	0.362319	0.362319
DR	1582.921	1601.129	1565.198	1588.405	1594.926	1598.327	1595.938	1605.117	1608.325
SNR	–	0.860958	1.011175	1.121636	1.198259	1.423725	1.571612	1.691983	1.78389
DL (ppm)	–	7.046411	6.306339	5.633005	5.397402	4.558676	4.171898	3.846089	3.690352
SR (nm)	–	2.113923	1.885595	1.652348	1.535561	1.292385	1.179256	1.087482	1.031453
χ (nm)	–	0.387165	0.657101	0.871742	1.055234	1.770005	2.398104	2.970874	3.481778

Here, *λ*_*TP*_ defines the position of TP resonance mode and Δ*λ*_*TP*_ describes the shift in the position of TP resonance peak due to the change in ethyl butanoate concentration (Δ*C*).

The investigated results in Table 1 clarify that our sensing tool could be of significant interest through the detection of ethyl butanoate. Herein, as investigated in Table 1, we have observed some changes within the parameters that support the performance of the suggested sensor. Based on the aforementioned Eqs. (10a–10i), these parameters are strongly depending on the characteristics of the TP resonance such as its position and FWHM. As the gas concentration changes, the optical path length of the incident light will be changed due to the change in the refractive index of the cavity layer. Therefore, the characteristics of the TP resonance such as its position and FWHM are strongly affected. Meanwhile, the performance of the proposed sensor in the vicinity of sensitivity, detection limit, the figure of merit, resolution, etc. will be affected as obtained through Table 1. However, we believe that these changes could be acceptable and have not led to a significant effect on the performance of the suggested sensor. In this regard, the proposed sensor provides a high sensitivity of 0.3 nm/ppm for just a concentration change of 5 ppm, we think this is a superb sensitivity compared with the previous literature. Meanwhile, the sensitivity does not decrease sharply with concertation, since it decreased to the value of 0.2795 nm/ppm for the entire range of concentrations (0–100 ppm). Surely this means that the proposed sensor has a stable sensitivity versus the different concentrations. Moreover, the reduced sensitivity (FM) which is preferred for experiments achieved remarkable values (0.10989 ppm^−1^– 0.101268 ppm^−1^) for the concertation range 0–100 ppm. The QF values are brilliant for all concentrations (the lowest one above the value of 968). QF determines the sharpness of the resonant dips. In the same manner, the sensor has a very good resolution represented by the low values of the FWHM (less than 2.86 nm), which indicates the high resolution of all resonant dips as well for any concentration value. In addition to that, the detection limit is a very important parameter determining how the sensor can detect the smallest changes in the concentration, here, the sensor has a detection limit in the range of 7–3 ppm for the concentration range of 0–100 ppm. The detection accuracy of the sensor ranges between 0.358423 nm^−1^ and 0.362319 nm^−1^. This means the sensor distinguishes between the much closed dips of an interval of 0.3 nm^−1^. The values of the signal-to-noise ratio are considered small ratios (They are ranging between 0.8 and 1.7), which means that the strength of the signal is more than the accompanying noise. To sum up, we can see that the sensor provides high performance for all parameters. Finally, in Table 2, we emphasize this high performance by making a comparison with previously published sensors of the same type.

**Table 2:** The sensitivity of our designed sensor in comparing with its counterparts in the related previous photonic sensors

References	Sensitivity (nm / RIU)
Ahmed and Mehaney (2019)	5018
Zaky et al. (2020)	190,000
Guan et al. (2011)	1179
Anamoradi and Fasihi (2019)	575
Huang et al. (2017)	850
Sansierra et al. (2019)	70
Das et al. (2015)	970
Qian et al. (2016)	450
Sharma et al. (2020)	9615
Our design	260,486

## Conclusion

To sum up, we have theoretically introduced a simple, efficient, and novel 1D PCs sensor for monitoring the concentration of ethyl butanoate in the dry exhaled breath. In particular, an efficient method for the detection of ethyl butanoate could be promising for accurate and rapid diagnosing of COVID 19. The main idea is depending on the inclusion of a cavity layer in which the dry exhaled breath can spread between the 1D PCs and a thin layer of Au. The numerical results show the appearance of a TP resonance peak through the reflectance spectrum of our structure. Such a peak provides very high sensitivity toward any variation within the characteristics of the cavity layer. In this context, the sensitivity could receive over 0.3 nm for a 1 ppm variation of ethyl butanoate in the dry exhaled breath. Moreover, the designed sensor provides good values of detection limit, detection accuracy and sensor resolution that could receive 7 ppm, 0.36 nm^−1^ and 2 nm, respectively. We believe that our designed sensor could be efficient in the monitoring and detection of many different gases.

## Data Availability

The data that support the findings of this study are available from the corresponding author upon reason-able request.
